# Policy and practice impacts of applied research: a case study analysis of the New South Wales Health Promotion Demonstration Research Grants Scheme 2000–2006

**DOI:** 10.1186/1478-4505-11-5

**Published:** 2013-02-02

**Authors:** Andrew J Milat, Rachel Laws, Lesley King, Robyn Newson, Lucie Rychetnik, Chris Rissel, Adrian E Bauman, Sally Redman, Jason Bennie

**Affiliations:** 1 Centre for Epidemiology and Evidence, NSW Ministry of Health, Locked Mail Bag 961, North Sydney, NSW, 2059, Australia; 2 School of Public Health, University of Sydney, Level 2, Medical Foundation, Building, K25, Sydney, NSW, 2006, Australia; 3 Sax Institute, Sydney, Level 2, 10 Quay, St Haymarket, NSW, 2000, Australia

**Keywords:** Government, Health promotion, Intervention research, Policy

## Abstract

**Background:**

Intervention research provides important information regarding feasible and effective interventions for health policy makers, but few empirical studies have explored the mechanisms by which these studies influence policy and practice. This study provides an exploratory case series analysis of the policy, practice and other related impacts of the 15 research projects funded through the New South Wales Health Promotion Demonstration Research Grants Scheme during the period 2000 to 2006, and explored the factors mediating impacts.

**Methods:**

Data collection included semi-structured interviews with the chief investigators (n = 17) and end-users (n = 29) of each of the 15 projects to explore if, how and under what circumstances the findings had been used, as well as bibliometric analysis and verification using documentary evidence. Data analysis involved thematic coding of interview data and triangulation with other data sources to produce case summaries of impacts for each project. Case summaries were then individually assessed against four impact criteria and discussed at a verification panel meeting where final group assessments of the impact of research projects were made and key influences of research impact identified.

**Results:**

Funded projects had variable impacts on policy and practice. Project findings were used for agenda setting (raising awareness of issues), identifying areas and target groups for interventions, informing new policies, and supporting and justifying existing policies and programs across sectors. Reported factors influencing the use of findings were: i) nature of the intervention; ii) leadership and champions; iii) research quality; iv) effective partnerships; v) dissemination strategies used; and, vi) contextual factors.

**Conclusions:**

The case series analysis provides new insights into how and under what circumstances intervention research is used to influence real world policy and practice. The findings highlight that intervention research projects can achieve the greatest policy and practice impacts if they address proximal needs of the policy context by engaging end-users from the inception of projects and utilizing existing policy networks and structures, and using a range of strategies to disseminate findings that go beond traditional peer review publications.

## Background

Public funds are expended on health research in large part to lead to improvements in policy [1-3], practice, resource allocation, and ultimately, the health of the community [4,5]. However, the transfer of new knowledge from research into practice continues to be far from optimal [2,6,7]. It is widely recognized that increasing the impact of research on policy and practice is likely to require many different strategies, including the development of research-policy partnerships, better summaries of evidence and more research-receptive policy and funding agencies [8,9]. It is also increasingly acknowledged that studies designed to evaluate the impact of interventions to improve health (intervention research) can inform subsequent intervention-specific policy and practice [10]. However, only a relatively small proportion (between 10-23%) of primary research funded by public health agencies or published in the peer reviewed literature is intervention research [11,12].

Little is known about the nature and mechanisms that underlie the influence of intervention research on health policy or practice. In fact, there are no agreed systematic approaches for measuring such impacts [13]. Traditional indices of research productivity relate to numbers of papers, impact factors of journals and citations. These metrics are widely used by research granting bodies, although they do not always relate well to the ultimate goals of applied health and medical research [14-16]. The emerging literature on research impact [17-19] highlights its complex, non-linear, unpredictable nature, and the propensity, to date, to count what can be easily measured, rather than measuring what “counts” in terms of significant, enduring changes [14].

A recent systematic review of approaches to assessing research impacts by Banzi *et al*. [13] identified 22 reports included in four systematic reviews and 14 primary studies. These publications described several theoretical frameworks and methodological approaches (for example, bibliometrics, econometrics, interviews, *ad hoc* case studies) to measuring research impacts, with the “payback model” as the most frequently used conceptual framework [19]. Based on this review of existing models, Banzi *et al*. differentiated five broad categories of research impacts: i) advancing knowledge; ii) capacity building; iii) informing decision-making; iv) health benefits; and, v) broad socio-economic benefits.

To date, most primary studies of research impacts (‘impacts research’) have been small scale case studies; and there has been no comprehensive assessment of impacts and their mediators across any single applied research funding scheme. The New South Wales (NSW) Health Promotion Demonstration Research Grants Scheme (HPDRGS) was designed by NSW Ministry of Health, Australia, in response to a paucity of evidence on large-scale intervention effectiveness across prevention policy priorities. The specific aims of the scheme are to fund applied research that builds the evidence-base for health promotion policy and practice; develop partnerships and build capacity for health promotion research between health districts, universities and organizations outside of the health sector. This paper reports on a exploratory case series analysis of all 15 projects funded under the HPDRGS during the period 2000 to 2006, to determine their subsequent policy and practice impacts (the ‘what’) and to explore the forces and factors influencing these impacts (the ‘how’ and ‘why’).

## Methods

**Figure 1:**
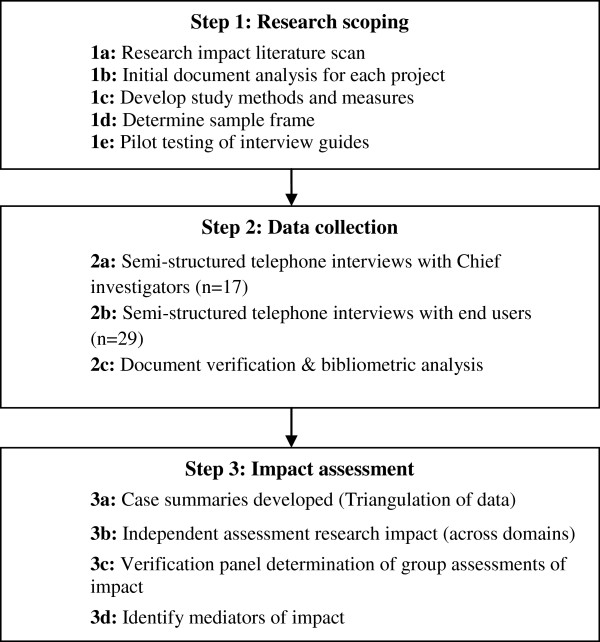
Overview of study methods and key steps in the research process

At the commencement of the study in January 2012, 15 projects funded during the period 2000 to 2006 had been completed for at least twenty-four months and most (n = 12) for longer than four years. This period was selected to balance the time required for evidence of impact to become manifest, against the potential accuracy of recall by respondents. A case study approach was used to explore if, and in what ways, research projects were used to influence policy and practice, and to identify the key factors (how and why) which influenced their use. Case study methods are appropriate for answering ‘how’ and ‘why’ questions when the phenomenon of interest (in this case, applied research) is embedded within a real-life context (policy and practice environment) [20]. Due to the diversity of projects under consideration, this case series included a number of different methods (Figure 1). The study was approved by the University of Sydney Human Research Ethics Committee and all participants gave written informed consent to take part in the study.

### Step 1 Research scoping

After considering the ‘research impact’ literature, we adapted the conceptual framework by Banzi *et al*. [13] as its domains aligned very closely with the objectives of the HPDRGS; with the five broad impact domains collapsed into four, as follows: i) Advancing knowledge and research related impacts (peer review articles, impact on research methods, better targeting for future research); ii) Capacity building (development of research capacity of staff, students, others); iii) Informing policies and product development (policy, guidelines, products, intervention development); and, iv) Health, societal and economic impacts (health status, social benefits, shift in knowledge, attitudes, behaviors, social capital, macroeconomic impacts, etc.). We combined categories four and five of Banzi’s framework (health benefits and broad socio-economic benefits) as pilot testing suggested they were both distal and inter-related and would be more efficiently described together.

Interview protocols for chief investigators (CI) and nominated end-users were guided by the adapted Banzi impact categories, lessons from the ‘research impacts’ literature scan and questions arising from a preliminary review of available documentation for the 15 projects. The interviews were then piloted with a CI and an end-user of two intervention research projects of commensurate size not funded through the HPDRGS.

### Step 2 Data collection

#### Semi-structured telephone interviews

The CIs were invited by email to participate in the study, with non-responders sent a reminder email after one week and then followed-up by telephone up to three times. Participating CIs were asked to nominate up to three end-users, defined as individuals who could provide a perspective on how the project had influenced policy, practice, organizational development, further research or in applications such as guidelines or teaching materials. CIs were encouraged to identify end-users from a range of sectors in which impacts occurred. These end-users were approached by email, using the same contact and follow-up procedure as CIs, to participate in an interview exploring how the project and its findings had been used from their perspective. CIs were typically university based academics or health service managers/researchers with joint appointments. While end-users were most frequently current or former policy makers, health service managers and practitioners.

Telephone interviews were conducted by an experienced research officer (RN) who has a good working knowledge of disease prevention, intervention research, and related policy and practice contexts; and was independent of the CIs and end-users. Interviews were digitally recorded with participants’ permission. Both CI and end-user telephone interviews explored perspectives on the overall impacts of individual projects, asked about specific impacts in relation to each of the four categories, and identified factors contributing to such impacts, or lack thereof. The following list outlines a summary of the telephone interview topic guides for CIs and end-users.

**Table 1:** **Project characteristics**, **key implications and dissemination methods used for HPDRG projects 2000**-**2006**

**Project title (year)**	**Funding A$**	**Study design**	**Intervention**	**Key outcome variables(statistical significance)**	**Key implications**	**Dissemination methods**
Making Connections (2000)	10,000	Action research	Community development	None (no comparator)	Goal of developing an early intervention program was not able to be achieved.	Report
Nutrition Practices in Youth Housing (2000)	38,100	Literature review and formative assessment	Nutrition standards	Improved nutritional practices (no comparator)	Provided input into the nutritional policy process for standards and accreditation of youth housing services, but was success limited by the political and institutional context.	Report, conference presentations, policy briefing
Reducing Smoking in Mental Health Units (2000)	38,449	Mixed methods (clinical audit, in depth interviews, surveys)	NRT, counseling with follow-up & organizational change	Offer and acceptance of NRT (no comparator)	Demonstrated NRT is acceptable to many staff and patients of mental health units and important for managing nicotine dependence in the context of a Smoke Free Workplace Policy. Identified key organizational and cultural barriers to practice change.	Report, conference presentations, presentation to local networks
Rural Hearing Conservation (2000)	17,670	Mixed methods (screening, mail survey)	Hearing screening and education	Hearing conservation behavior(no comparator)	Confirmed the extent of hearing loss in farmers and salience in rural communities. Recommended that the Rural Hearing Conservation Program be continued and, where possible, expanded.	Report, summaries, conference presentations, peer-reviewed papers, policy briefings, website
Secondary Prevention in Patients with CVD (2000)	239,295	Cluster RCT	Mailed information packages for patients, general practitioners and combined intervention	Medication use (NS) Behavioral risk advice [exercise, diet & smoking cessation] (NS) except for physical activity (*P* = 0.04) in patient intervention stream	There is insufficient evidence upon which to make a recommendation that information only interventions should be incorporated into the routine practice of CVD registers.	Report, conferences, presentations to local clinical groups
Tai Chi and Falls Prevention for Older People (2000)	274,384	RCT	Tai chi exercise	Falls incident rate (*P* = 0.008)	Participation in weekly tai chi classes prevents people falling multiple times and improves balance in community dwelling older people. As the trial used existing community facilities it provided a model for an effective and sustainable public health intervention.	Report, conference presentations, peer-reviewed papers, thesis, presentations to practitioner networks
				Balance:		
				Sway on floor (*P* = 0.04)		
				Sway on foam mat (*P* = 0.001)		
				Lateral stability (*P* = 0.04)		
				Coordinated stability (*P* = 0.005).		
Mental Health First Aid Training (2002)	178,432	Cluster RCT & qualitative evaluation	Mental health literacy training	Correct diagnosis (*P*=0.001) Help offered to person with mental health problem (*P* = 0.031)	The training course produced positive changes in knowledge, attitudes and behavior when given to members of the public by instructors from a local health service. Showed strong promise a strategy in broader mental health promotion and workforce development initiatives.	Report, conference presentations, peer-reviewed papers (open access), policy briefings, training, political advocacy, websites, media
Safer Streetscapes for Older People (2002)	179,609	Mixed qualitative methods	Fall risk assessment and capital works	Environmental and policy change (no comparator)	Qualitative consultative methods employed elicited information from older people that can be used to advocate for changes to the streetscape.	Report, conferences, council briefings
Treatment of Nicotine Dependant Inpatients (2002)	249,970	Quasi-experimental design	Smoking care provision	Smoking care outcomes:	Incorporation of smoking care intervention strategies into routine clinical and organization performance management and accreditation processes has the potential to facilitate widespread NRT provision in hospitals.	Report, conference presentations, peer-reviewed papers, thesis, presentations to policy, practitioner & university networks, media
				Smoking status identified (NS)		
				Management of smoking discussed (*P* = 0.01)		
				Offered NRT (*P* <0.001);		
				Provided NRT (*P* <0.01)		
				Provided written resources (*P* <0.01)		
				Provided post-discharge NRT (*P* = 0.03).		
				Monitored withdrawal (NS)		
				Advised discharge support (NS)		
Pedometers in Cardiac Rehab (2004)	200,000	RCT and focus groups	Pedometers, step calendar, and behavioral counseling and goal setting	Physical activity:	A pedometer based intervention can be offered as an effective and accessible option for those who do not attend cardiac rehabilitation to increase their physical activity levels. This intervention could also be promoted as an important adjunct to existing cardiac rehabilitation programs.	Report, conference presentations, peer-reviewed papers, thesis, local presentations
				Total physical activity time (*P* = 0.044)		
				Total physical activity sessions (*P* = 0.016)		
				Walking sessions (*P* = 0.035)		
				Psychosocial:		
				Cognitive self-management strategy use (*P* = 0.001)		
				Psychological distress (*P* <0.001)		
Reducing Falls Injuries within Aged Care (2004)	300,000	Cluster RCT	Multi-strategic best practice falls prevention strategies	Facility level:	It was difficult to change the culture within residential aged care and particularly of the visiting medical officers. It seems unlikely that any sustained reduction in hip fractures in residential aged care facilities can be obtained without outside support.	Report, conference presentations, peer-reviewed papers, presentations to policy & practitioner networks
				Fall risk assessment (*P* = 0.002)		
				Monthly number of falls (NS)		
				Any fracture (NS)		
				Hip fracture (NS)		
				Death (NS)		
				Individual level:		
				Hip fracture (NS)		
				Death (NS)		
Walk-to-School (2004)	257,000	Cluster RCT	Education, travel access guides, environment change	Student mode of travel reported by students:	No clear pattern in the results, due to the high variation in the percentages of students in the intervention and control schools who changed their travel mode. The research identified the strong influence of the parent’s journey to work on their child’s journey to school. The project contributed to methodological development in this field of research.	Report, conference presentations, peer-reviewed papers, presentations to local councils, media
				Morning journey (NS)		
				Afternoon journey (NS)		
				Student mode of travel reported by parents:		
				Morning journey (NS), except walking trips in a usual week (*P* = 0.05)		
				Afternoon journey: (NS)		
Cycling Infrastructure and its Promotion (2006)	280,537	Quasi-experimental	Community engagement and social marketing	Sufficient activity to confer health benefit (NS)	There was no overall increase in the prevalence of cycling in the intervention area, and therefore there was no difference in overall levels of physical activity between the intervention and comparison areas. After adjusting for baseline levels of minutes riding, there was a significant increase in the total mean number of minutes riding in the intervention area compared with the comparison area.	Report, conference presentations, peer-reviewed papers
				Mean minutes of physical activity (NS)		
				Adjusted mean number of minutes riding (*P* = 0.039)		
Exercise to Prevent Falls After Stroke (2006)	292,708	RCT	Group-based physiotherapist-led exercise classes and advice	Falls incident rate (NS)	No overall effect on falls, exploratory analysis however did find a significant differential effect of the intervention according to baseline walking speed. The intervention was more likely to prevent falls in faster walkers.	Report, conference presentations, peer-reviewed papers, policy briefings, newsletters, practitioner networks, political advocacy
				Falls based on faster initial walking speed (*P* = 0.03)		
Smoking Cessation in Indigenous Communities (2006)	290,200	Action research (mixed qualitative and quantitative methods)	Subsidized nicotine replacement therapy and weekly support sessions with case manager	Provision and use of NRT (no comparator) 12 month quit rate (no comparator)	Twenty-four percent of Smokers Program participants remained smoke-free at a minimum of 12 months after Program completion. Program prompted people to attempt quitting and provided opportunities for health workers to talk about smoking and smoking-related illness with their clients.	Report, conference presentations, local seminars, Aboriginal health worker research forums
**TOTAL**	**2**,**846**,**354**					

NS: No significant between-group differences; NRT: Nicotine replacement therapy; RCT: Randomized controlled trials.

Semi-structured telephone interview topic guide: Investigators and End users

•Recall of research aims, key finding and implications

•Dissemination process (how, factors influencing the dissemination process)

•Interface with end users – how research team worked with potential end users (investigators only)

•Interface with researchers – how were end users involved in the research project, how did they hear about the findings (end users only)

•Overall impact – how have the findings been used

•Specific impacts – capacity building, partnerships, policy and product development, health and other sector impacts, societal and economic impacts

•Circumstances surrounding the use of the findings, or limited impact of the findings

•Evidence of impacts- documentary sources

•Nomination of end users (investigators only)

#### Bibliometric analysis

A bibliometric analysis was also undertaken in Scopus in April-June 2012 to examine the total and mean number of citations (excluding self-citations) for all peer review publications arising from each project. Project reports were located and examined to document key project findings. Respondents were also asked to provide copies of additional documentary sources as evidence of how project findings had been used, such as policy documents, briefs, reports and curriculum materials. Additional searches of the grey literature were undertaken to corroborate documentary evidence of impacts reported in the interviews. Documentary evidence was compiled by the research officer (RN) and checked by two other authors (AJM and JB).

### Step 3 Impact Assessment

#### Data synthesis and verification panel

Interview and document data were collated and triangulated in ‘case summaries’ by two authors (AJM and JB) and reviewed for accuracy by the research officer who conducted the interviews (RN). Case summaries for each project included: i) key research findings and implications; ii) the perspectives of CIs and end-users on how project findings had been used and key factors influencing use, including illustrative quotations; iii) bibliometric analysis; iv) documentary evidence of impacts; and, v) notes and observations made during CI and end-user interviews. The coding framework for analyzing these case summaries was based on impact domains, contextual information and key factors influencing research use.

A verification panel was established to review and assess the collated case study material, and provide an overall assessment of the policy and practice impact of each of the 15 projects. Our approach was adapted from the RAND/UCLA (University of California, LA, USA) appropriateness method [21,22]. This systematic consensus method has been widely used to derive expert consensus on clinical indications, quality improvement and assessing effectiveness of health networks [23,24].

The verification panel was made up of eight members of the research team: a mix of senior academics and policy makers, including international experts in the field of applied population health research. Case summaries of each project were independently assessed by panel members across the four impact domains and overall impact. Assessments were made using a nine point scale: 1 to 3 ‘limited impact’; 4 to 6 ‘moderate impact’; and 7 to 9 ‘high impact’. Judgments of overall impact took into account the four impact domains as well as: size of the project and level of funding; time since project completion; potential sustainability of the impact; and, research and implementation challenges that were addressed in creating the impact. Individual ratings were compiled and discussed at a verification panel meeting held in August 2012, where consensus was reached on overall impact assessments for all 15 studies. The panel also identified a number of key influences on policy and practice impacts across projects, which were further explored by a final analysis of the data to describe ‘how’ and ‘why’ projects were impactful or not.

## Results

### Project characteristics

Between 2000 and 2006, fifteen projects were funded across a broad range of topics, using a range of study designs, most commonly RCTs (n = 7), mixed methods (n = 5) and quasi-experimental designs (n = 2) (Table 1). Most projects employed a mix of qualitative and quantitative methods (n = 13). Funding ranged from 10,000 to 300,000 Australian dollars per project. Projects were most commonly implemented in community (n = 9) and health services (n = 5) settings in both rural (n = 8) and metropolitan areas (n = 7).

**Table 2:** **Interview sample**, **research outputs**, **means of independent assessment and panel assessment of overall impact for projects funded between 2000 and 2006**

	**Interviews**		**Research outputs**			Mean independent assessment of impacts*					Panel assessment of impact*
**Project(year)**	**CI(n)**	**EU(n)**	**Conferences(n)**	**Papers(n)**	**Citations(n)**	**Advance knowledge (mean)**	**Capacity building (mean)**	**Policy &practice** (mean)	**Health,social,economic (mean)**	**Overall (mean)**	**Group overall**
**Ranked by Group Overall impact**											
Mental Health First Aid Training (2002)	1	3	n/a	4	66	**8**	**7**	**8**	*6*	**8**	**8**
Tai Chi and Falls Prevention for Older People (2000)	1	3	7	2	77	**8**	**7**	**7**	*6*	**7**	**7**
Treatment of Nicotine Dependant Inpatients (2002)	1	4	3	7	31	*6*	*6*	*6*	*5*	**7**	**7**
Rural Hearing Conservation (2000)	1	2	4	4	14	*6*	*6*	**7**	*4*	*6*	*6.5*
Smoking Cessation in Indigenous Communities(2006)	1	1	4	0	-	*4*	*5*	*6*	*4*	*5*	*6.6*
Pedometers in Cardiac Rehab (2004)	1	2	4	2	6	*6*	*5*	3	2	*5*	*5*
Exercise to Prevent Falls After Stroke (2006)	1	2	2	3	3	*5*	*4*	*5*	3	*5*	*5*
Walk-to-School (2004)	1	1	n/a	6	44	*6*	*4*	*4*	2	*4*	*4*.*5*
Reducing Falls Injuries within Aged Care (2004)	2	2	2	1	1	*4*	*4*	*5*	3	*5*	*4*.*5*
Reducing Smoking in Mental Health Units (2000)	1	2	0	0	-	3	3	*4*	2	*4*	*4*
Cycling Infrastructure and Its Promotion (2006)	1	3	5	4	23	*5*	*4*	*4*	3	*4*	*4*
Secondary Prevention In Patients with CVD (2000)	1	1	0	0	-	3	*4*	3	2	3	2
Nutrition Practices in Youth Housing (2000)	2	2	4	0	-	2	1	1	1	2	2
Safer Streetscapes for Older People (2002)	1	-	1	0	-	2	3	3	2	3	2
Making Connections (2000)	1	1	0	0	-	1	2	1	1	1	1
**Total(mean)**	**17**	**29**	**36**	**27**	**265**	**(4)**	**(4)**	**(5)**	**(3)**	**(5)**	**(4.5)**

*Key: 1–3: ‘limited impact’; *4*–*6*: moderate impact; **7**–**9**: high impact.

### Semi-structured interviews and panel impact assessments

A total of 46 interviews were conducted (Table 2), with CI interviews (mean duration: 53.3 mins; range: 38 to 97 mins) lasting longer than end-user interviews (mean duration: 40.0 mins; range: 19 to 81 mins). The response rate for CIs was 70.8% and 74.4% for end-users.

**Table 3:** How projects and their findings informed policy and practice

**Key Impacts**	**Illustrative Quotes**
***Policy Impacts***	
**Agenda and priority setting,** e.g., attracting funding to the issue of interest, identifying priority groups and settings for intervention.	“[*evaluation findings*] *I think it did have an effect because we were able to promote the issue of hearing loss and the need for protection and in a way that we hadn*’*t been able to before and it*’*s just become a more important issue*.”(*EU1* - *Rural hearing*)
**Informed policy debates,** e.g., data used in briefings with health ministers, inform parliamentary debates, and met with senior bureaucrats.	“[*attended*]…*the Victorian State Parliament inquiry into mental illness and work*, *and talked about mental first aid*, *and the politicians were very enthused about it all*…*And we*’*ve certainly met with individual politicians*, *and individual public servants*. (*CI* - *Mental Health First Aid* )
**Informed policy planning,** e.g., identifying areas for investment in tai chi for older people and smoking cessation brief intervention.	“…*knowing that tai chi could be effective in resisting falls*, *means that it*’*s something that we can promote and recommend for falls prevention*.” (*EU1* - *Tai Chi and Falls*)
**Directly underpinned new policy,** e.g., provision of mental health first aid in human service agencies across Australia, inclusion of physical activity in the NSW Falls Prevention Policy 2007–2010, importance of developing specific measures to reduce smoking in mental health units.	“…*in the early days where people were struggling with implementation of the smoke*-*free workplace policy*, *sort of just showed a way for the people and a comprehensive approach*…*And to be able to demonstrate that we could affect change across whole hospitals was really an important thing to be able to say that it can be done*. “ (*CI* - *Nicotine Dependent Inpatients*)
**Used to support existing policy,** e.g., importance of smoke free environments in mental units, supported the implementation of NSW Smoke-free Workplace Policy and proved that the strategies proposed in the NSW Guide for Nicotine Dependent Inpatients could be implemented.	“…*it sort of reinforced the understanding that mental health services are a specific and special case*. *And that we needed to make sure that we had specific guidelines and that there was more buy in from consumers and psychiatrists and all of the other stakeholders*.” (*EU2* - *Reducing Smoking in Mental Health Units*)
**Evaluated existing programs,** e.g., Rural Hearing Conservation.	
***Practice Impacts***	
**Informed organizational development in the health sector,** e.g., provided that standardized approach provision of smoking cessation advice in health services.	“*So Mental Health First Aid is core business for a lot of people working in*, *what was typically a tertiary and mental health service*, *people providing clinical services*, *now there*’*s a big health promotion*, *early intervention strategy*…” (*EU2* - *Mental Health First Aid*)
**Lead to new intervention tool and resources,** e.g., standardized provision of tai chi, mental health literacy training, provision of exercise for stroke survivors, materials to support falls prevention in aged care facilities.	“*it*’*s provided a model of best practice that*’*s been able to be implemented really broadly*.” (*EU2* - *Falls and Aged Care*)
**Informed professional development for health staff**, **human service workers and fitness leaders,** e.g., smoking cessation brief interventions, provision of tai chi to older people.	“[*project officer*] *now has a PhD*, *she*’*s a lead researcher and program manager and developer in our organization and that really come out of the opportunity*. *If the funding had not been there to do that program of work*, *that wouldn*’*t have happened*…” (*EU1* - *Nicotine Dependent Inpatient*)
**Informed and supported existing health promotion programs,** e.g., Rural Hearing Conservation Program, Tai Chi for Older People.	
**Informed program planning,** e.g., choice of target groups and settings for intervention and availability of treatment programs.	

There was limited variation between panel members in their assessments of the overall impact of each project, and consensus on the final group overall impact assessment was achieved easily. Three studies were considered to possess ‘high’ overall impact (Tai Chi, Mental Health First Aid and Nicotine Dependent Inpatients), eight ‘moderate’ overall impact (Rural Hearing Conservation, Smoking Cessation in Indigenous Communities, Pedometers in Cardiac Rehab, Exercise to Prevent Falls after Stroke, Walk-to-School, Reducing Falls Injuries within Aged Care, Reducing Smoking in Mental Health, Cycling Infrastructure), while four were rated as ‘low’ overall impact (Nutrition Practices in Youth Housing, Secondary Prevention in Patients with CVD, Safer Streetscapes, Making Connections). Impact ratings across the adapted Banzi categories, as well as overall impact assessments for each project, are shown in Table 2.

**Table 4:** **Factors influencing impacts of research on policy and practice** (**across case studies**)

**Facilitators**	**Barriers**
***Nature of the intervention***	**Poorly defined interventions without a clear purpose and outcomes**
**Simplicity of intervention**. Easy to explain and has a clear rationale	“… *it was almost an impossible project and it was starting from no base*. (*EU1* – *Making Connections*)
**Capacity of intervention to be packaged and** ‘**agents**’ **trained in its delivery**	**Use of intervention approaches that are difficult to replicate in other settings and target groups**
“*It*’*s very structured*, *very organized* – *it comes with comprehensive teaching notes and instruction and people keep in contact* – *even though people aren*’*t employed by Mental Health First Aid Australia in Melbourne*, *they refer to*, *what I call the mother ship*, *on a regular basis and keep in close contact*…” (*EU2* - *Mental Health First Aid*)	
**Can be easily replicated and scaled**-**up**	
**Organization change approaches**	
“…*we developed* – *the policy compliance procedure an annual audit of the records of the patients who were on the ward at the day so you*’*ve got an annual reporting of whether procedures are being complied with*.” (*EU3* - *Nicotine Dependent Inpatients*)	
**Integration into usual practice**	
“*I think that really the key things are that the program was integrated into the core business of the service*…*the very fact that you have ongoing dedicated support from trained workers*, *that that*’*s clearly a key component of the success of the program*…” (*EU 1* - *Smoking Cessation in Indigenous Communities*)	
**Project aligned to the priorities of policy makers and practitioners with adaptations made over time to meet needs**	
“…*so it was really from someone in the* [*Department*]*head office making that remark that then led to the other project which was never what we envisaged but it was still a very good idea*.”. (*CI* - *Mental Health First Aid*)	
***Effective partnerships***	
**Partnerships formed through research projects led to deeper relationships and further policy driven research**	**Inability to form partnerships with key influencers and end**-**users**
““*It has influenced our research direction for my colleagues*… *it*’*s promoted a bit more inter*-*professional research opportunities*” (*CI* - *Stroke and Falls*)	“*So they did try and form different partnerships*, *but that*’*s a very fractured and continues to be a very fractured area to work in*.” (*EU* – *Making Connections*)
**Continuity and partnerships between researchers and end**-**users** from the inception of projects facilitated dissemination, ownership and use of the findings.	
“…*relationship between* [*Chief Investigator*] *and the Local Area Health Service*, *definitely strengthened*, *and I think that has been demonstrated by that second demonstration grant*.” (*EU2* - *Pedometer and Cardiac Rehab*)	
***Leadership and champions***	
**Multiplier effect of leadership** “*So I guess having champions in an area health service*… *just infects the whole system if you like because if one area is doing it rigorously from a research perspective and building on the research*.” (*EU4* - *Nicotine Dependent Inpatients*)	**No clear alignment with potential leaders in the field of interest who can advocate for project findings**
***Research quality***	
**High research quality and credibility**	**Poor research quality**
“… *based on those early trials*, *it gives you confidence to say*, *well we know it worked*.”(*EU3* – *Mental Health First Aid*)	“…*the clear finding to me was it should never have been funded*” (*EU* - *Making Connections*)
“ *And*, *also*, *the fact of having it published in the Peer Review Journal*…*in the Cochrane Review which I think that*’*s very influential that review in terms of setting the agenda for what kind of interventions will be funded in Australia and internationally in falls prevention*. *I think that*’*s really important*.” (*EU1*- *Tai Chi and Falls*)	**Projects findings did not provide definitive answers**, needed to be considered alongside a body of evidence about effective interventions. “…*you can see because it*’*s a kind of a mixed finding*, *so people think*, *oh*, *that*’*s too hard*” (*CI Walk to School*)
***Dissemination approaches***	
**Use of active dissemination strategies** such as discussion of findings at workshops between researchers and end-users. “*So it*’*s got a lot of dissemination through talks we*’*ve done all over the place*, *nationally and overseas*.... *A lot of it would be things like departmental seminars*” (*CI* - *Mental Health First Aid*)	**Findings not tailored to end**-**users needs**
	**Poor links with policy makers and practitioners networks** “*When I first read it*, *I thought I don*’*t know anything about this*.” (*EU* - *Making Connections*)
**A range of** ‘**knowledge transfer**’ **products produced**, e.g., short reports highlighting key findings and recommendations, well packaged project resources, websites, etc.	
“[*About publishing in open access journals*] *Anyone can go onto the Web and find it and get the full text of it for free*. *And we did that deliberately as a strategy because we wanted the findings available to anyone*… *And the website now is a major dissemination source and the report of this study is there on that website*.” (*CI* –*Mental Health First Aid*)	
**End**-**users acted as** ‘**knowledge brokers**’ facilitating dissemination of project findings within their sector “…*we had a*.... *busy email list there with a lot of sharing*, *a lot of questions*. *We realized that there was a real need for trying to skill up clinicians in how to work with people in addressing nicotine dependence*.” (*EU4* - *Nicotine Dependent Inpatients*)	
***Contextual issues***	
**Supportive policy context** for addressing the issues with the release of project findings fitting well with some policy cycles (Smoke Free Workplace Policy, Falls Prevention Policy)	**Political instability and poor timing** Frequent changes in health ministry positions, health service restructures and poor fit with some policy cycles.
“*they*'*d had a whole practical level of working with hospitals to try to get this stuff to happen*, *they were able to help us compose the performance criteria*.” (*EU3* – *Nicotine Dependent Inpatients*)	“*And*, *then*, *a lot of the restructuring within area health services and within the department*…*had an impact on getting the falls plan out*.” (*EU1* – *Tai Chi and Falls*)
**Mechanisms and structures in place to profile findings and implement recommendations**, e.g., Policy relevant forums involving key end-users	**Limited sector capacity and resources**, e.g., lack of funds to implement the findings in some sectors:
“*I have a link now with the Heart Foundation and the Heart Foundation and Stroke Foundation are now more closer working together*; *it*’*s largely come out of this work as well so the National Stroke Foundation with having round tables at the time of this project* ”(*CI* - *Falls and Stroke*)	“…*it*’*s not because we actually get funding*, *it*’*s just because we have a farm safety group here made up of a mix of farmers*, *Essential Energy*, *CWA*, *Department of Primary Industries and a few other groups that have literally kept this alive*.” (*EU1* - *Rural Hearing Conservation*)
**Ensuring good fit with organizational culture and ways of working**	
“…*in environments that have already got established sort of chain of command and specific behaviors and expectations*, *and then go in and start telling people what to do*. *It doesn*’*t work*. *And often I went in and did a lot of it in the beginning to show them that it wasn*’*t a great deal of work*.” (*EU3* - *Nicotine Dependent Inpatients*)	
**Alignment with policy priorities**	
“…*when these clinical practice guidelines came out*, *there was greater take up*… *because they had already done the ground work*.” (*EU3* - *Nicotine Dependent Inpatients*)	
**Confluence of events**	
“*When it went to the United States*, *a very important thing*… *Virginia Tech massacre*. *And there*’*d been this student who had a mental illness and nobody did anything about it*…*that was then an external event that had an influence on its spread to America*.” (*CI* - *Mental Health First Aid*)	

### Advancing knowledge

Projects sought to advance knowledge using a variety of dissemination methods including reports, peer-reviewed papers, conference presentations, theses, presentations to stakeholder groups, political advocacy, training, websites and the media (Table 1). Peer reviewed papers generated by projects ranged from 0 to 7, with a mean of 10 citations per paper (range: 0 to 73); 96% of these citations came from six projects, which were rated as high or moderate impact studies. The two projects independently rated as having a ‘high impact’ were the Tai Chi and Mental Health First Aid projects. The studies with the highest citations were effective interventions which provided novel results for the field of interest. High and moderately impactful projects were all managed by experienced researchers, and high quality publications were produced despite equivocal findings in some instances. All of the studies with low impact on advancing knowledge had null study results, no publications and were mostly led by inexperienced researchers and practitioners.

### Capacity building impacts

Both CIs and end-users indicated that capacity building occurred through staff development, partnership building and follow-on research funding. For many end-users, projects provided opportunities to develop their research skills and partnerships with researchers. Researchers of the two projects with high capacity building impact, Tai Chi and Mental Health First Aid, consistently stated that projects helped them to build their own research capacity and partnership networks, enabling them to build enduring connections to policy and practitioner networks from which a body of research emerged. A number of CIs and end-users of high and moderately impactful projects spoke of projects as a place where future research and service ‘leaders’ were trained.

### Policy and practice impacts

In terms of policy impacts, end-user respondents from high and moderate impact studies reported using research to inform agenda setting and policy debates. Project findings also informed policy planning, and in some cases underpinning elements of new policies in health services. At the practice level, high and moderate impact projects were reported as being used to inform program planning across a range of sectors. In the health sector a number of projects (Treatment of Nicotine Dependant Inpatients, Reducing Smoking in Mental Health; Smoking Cessation in Indigenous communities) resulted in substantial practice changes in the provision of smoking cessation advice and nicotine replacement therapy in health services. A number of projects also informed organizational development, where interventions were integrated into core business of health services. One such study (Tai Chi) led to a much more standardized provision of falls prevention interventions in community setting across large parts of the state of NSW.

In some cases, high impact research provided retrospective support and rationale for existing health promotion programs, such as the NSW Rural Hearing Conservation Program. Overall, practice impacts appeared to largely flow from policy impacts. For example, the policy focus on tobacco control in hospital settings contributed to the development of new practice resources and professional development for smoking brief intervention in hospitals, as well as in mental health units. A summary of how projects and their findings influenced policy and practice and illustrative quotes derived from interviews are provided in Table 3

### Broader health, economic and societal impacts

None of the projects were independently assessed as ‘high impact’ in the health, societal and economic impacts domain, with a mean ‘moderate’ rating being the highest achieved for the Rural Hearing Conservation Program, Tai Chi, Mental Health First Aid, Treatment of Nicotine Dependant Inpatients, Falls in Aged Care and Smoking Cessation in Indigenous Communities programs.

### Factors influencing policy and practice impacts: the ‘how’ and ‘why’

Examination of patterns differentiating high, moderate and low (overall) impact intervention research at the verification panel and further thematic analysis of interview transcripts identified six key factors that particularly contributed to these impacts. A summary of these factors and illustrative quotes derived from the interviews are collated in Table 4.

### Nature of the intervention

All of the studies considered to have high policy and practice impacts (Tai Chi, Mental Health First Aid and Treatment of Nicotine Dependent Inpatients) also had moderate to high ratings for advancing knowledge and strong research outputs. However, a number of studies that achieved moderate to high ratings in advancing knowledge, and demonstrated strong research outputs (journal papers and citations) failed to achieve high levels of real world policy and practice impacts, namely the Walk to School and Cycling Infrastructure programs. Data suggest that these projects lacked definitive results and a clear agency with policy responsibility, where policy makers could advocate for their replication and expansion. In addition, the complex and inter-sectoral nature of these interventions, that require environmental and cultural change to achieve intended outcomes made them difficult to readily replicate or scale-up.

Further examination of studies with low and moderate impact also highlighted a number of barriers to applying findings, including not producing clear results indicating effective action, interventions and outcomes that were hard to explain, and no consideration of how effective interventions could be scaled-up for population level implementation. The majority of high impact projects effectively packaged intervention materials and tapped into readily available workforce to expand program reach. To illustrate, in the space of nine years the Mental Health First Aid program has been scaled-up using a ‘train the trainer’ model to the point where it has reached 1% of the Australian Population [25].

It is interesting to note that though high and moderately impactful projects generally received larger amounts of funding, this alone was not always related to impact. The Rural Hearing Conservation project evaluated an existing program with minimal resources (A$17,670), providing a high return on investment in terms of policy and practice impacts. Also, a number of the least impactful projects received large amounts of funding.

### Leadership and champions

Highly impactful projects all displayed strong networks of leaders and champions who advocated for further adoption of interventions into policy and practice. These individuals were found to promote the benefits of the intervention across a variety of stakeholder groups including, politicians, media, policy makers and the general public, as well as relevant professional and academic networks. Champions included CIs, end-users and chief executive officers of organizations within which interventions were trialed, as well as intervention service providers who had a commercial interest in expanded program delivery.

### Effective partnerships

For the majority of high and moderately impactful studies, partnerships between end-user groups and the CIs existed from the inception of the projects. The analysis showed that in many cases ongoing relationships provided the continuity and mechanisms for project findings to be disseminated and considered, and for end-user groups to become engaged in formulating the key policy recommendations and wider dissemination processes. These partnerships also allowed researchers to tap into prevailing policy priorities and were considered an important contributor to their capacity to undertake further priority-driven research in partnership with end-users.

### Dissemination approaches

Impactful projects consistently used active dissemination strategies, such as discussion of findings at workshops between researchers and end-users, as well as dissemination of findings through established policy and practitioner networks. These projects also developed ‘knowledge transfer’ products, such as short reports highlighting key findings and recommendations and packaged project resources/materials, making them available on websites for broader use. Some high impact studies intentionally published findings in open access journals, as a way of disseminating project findings to a broader audience of end-users. Analysis of low impact studies indicated that they, for the most part, gave little consideration to dissemination processes and, in a number of instances, offered no analysis of broader policy implications of project findings.

### Perceived research quality

Research quality was consistently cited by a number of end-users of high impact projects as an important consideration in their use of research findings. However, end-users also stated that decisions to change or modify policy or practice were informed by the ‘body of evidence’, rather than findings of single studies.

### Contextual factors

Among the numerous contextual factors identified as potential facilitators to the application of research findings, one of the most influential was the prevailing policy ‘zeitgeist’. CIs and end-users of high impact projects spoke of a study’s ability to provide a potential solution to a pressing policy problem. So much so, that some projects gained momentum through external factors, such societal events and parliamentary inquiries, that focused community and political attention on issues for which research could provide a response (such as Mental Health First Aid). For low impact studies, some of the key impediments to applying the findings comprised circumstances where researchers did not have capacity to establish and maintain links with policy makers or with the current policy priorities.

## Discussion

While a growing number of studies have examined impacts of research [13] and research funding [26], this is the first study to document the impacts of a policy-driven applied research funding scheme. This analysis of research impacts indicates that some, but not all, of the intervention research funded through the HPDRGS achieved a wide range of tangible impacts across most domains. It is clear that the three projects with the highest overall impact ratings in this study had substantial impacts on advancing knowledge and capacity building, as well as policy and practice. However, some projects with substantial research impacts (papers and citations) yielded only minimal policy and practice impacts. This reinforces that traditional indices of research impact and researchers’ track record on publications and grants are not always an accurate guide to the policy and practice impacts of their research.

This case study analysis demonstrates the positive impact that intervention research funding can have on a range of policy and practice decisions, with findings used as a policy advocacy tool (to attract attention and funding to an issue), for priority setting (identifying areas and target groups for intervention), and to support and justify existing programs/approaches or identify the need for alternatives. In a number of instances project findings informed the early stages of policy development, when there had previously been a lack of definitive evidence about effective intervention approaches. We also found that research findings were used to directly underpin key elements of existing policies for falls prevention in older people, and tobacco control. In addition, findings were used to improve understanding of issues associated with implementing and assessing new interventions such as travel guides and other promotion of active transport.In many instances the use of project findings by practitioners reflected a need to act on state-wide policy imperatives. The introduction of the NSW policies on smoke free hospitals and falls prevention saw many practitioners tasked with developing local responses using relevant HPDRGS project resources. This highlights the value of having research funding aligned with state-wide policy.

It is clear from this analysis that many factors influence public health policy and practice, with evidence from an effective intervention study in itself generally not enough to shift the current approaches. Consistent with previous research [5], we found that findings of a single study were usually considered alongside a broader body of evidence about effective intervention approaches, as well as a consideration of the local context and timing requirements. In a seeming paradox, some studies that had null or equivocal results still achieved moderate policy and practice impact. This suggests that adoption of project findings into policy and practice are influenced by factors other than evidence of effectiveness. Closer review of these projects revealed that the introduction of state-wide policies and programs meant that practitioners adopted the available project materials, meeting an immediate practice need, even though studies were demonstrably not effective.

Further, we found that a range of contextual factors were critical in facilitating the use of the projects findings, which is in agreement with previous studies [27-31]. In particular, supportive policy contexts encouraged partnerships between researchers and end-users from the inception of projects, and where possible utilized existing structures (policy and practitioner networks, etc.) for communication. Tapping into existing policy and practitioner networks and processes appeared to enable researchers to build partnerships and trust with practice and policy ‘users’ and allowed better utilization of policy ‘windows of opportunity’. In some instances a confluence of events provided the right conditions for an intervention to be widely adopted into policy and practice. One such tragic event was the Virginia Tech massacre in the United States that highlighted the importance of mental health literacy and was thought to be a critical factor by the CI of this project in driving the early expansion of Mental Health First Aid in North America. All of the high impact projects were characterized by simple interventions that were well implemented, high quality research, champions to advocate and disseminate for adoption, as well as supportive contextual factors. The review of materials by the verification panel identified that an intervention’s capacity to be packaged and change ‘agents’ trained in its delivery was particularly important, as evidenced by the rapid expansion of Mental Health First Aid and Tai Chi across respective practice settings.

This study supports a growing body of evidence about the importance of embedding and linking research with broader strategic policy contexts. In a systematic review of 24 studies of the use of evidence by health policy makers, Innvaer and colleagues [28] found that personal contact, timeliness and relevance were the most commonly reported facilitators of research use. In our current study, impactful projects appeared to effectively engage key end-users groups, to ensure that projects were aligned to the interests and needs of such groups and to promote ownership of the findings and by doing so, increasing commitment to action. Most of the low impact studies had no such clear links with end-users or existing policy and practice networks.

Findings of this and other recent studies [31] also highlight the importance of the production of a range of dissemination products such as short reports, fact sheets and project resources, and their availability through websites, as well as publishing in open access journals to facilitate the use of the findings by end-users. There is increasing emphasis from funding agencies on making research evidence readily available [32,33]. Yet, recent studies of public health research suggest that most dissemination activity rarely goes beyond publishing academic papers, appears to be undertaken in an *ad hoc*, unfunded fashion, and that access to dissemination advice and support for researchers from funding agencies and academic institutions is lacking [34,35]. This study highlights the value of funding and systematically supporting a wide range of dissemination activities.

The excellent return on investment from the Rural Hearing Conservation Program Evaluation highlights what can be achieved with limited resources when research funding is well targeted. There appears to be merit in funding high quality evaluations of existing policies and programs. Increasingly, funding agencies require investigators to detail how their research impacts on policy and practice [36,37], and a growing number of theoretical frameworks for assessing impact have been proposed [16,38-41]. This study demonstrates the utility of the scoring and panel verification methods used for identifying and measuring proximal research impacts (advancing knowledge, capacity building and policy and practice impacts). The findings of this and other recent studies [31] suggest however, that the longer-term impacts (societal, health and economic) of a single study can be difficult to discern and attribute. The CIs, end-users and verification panelist all reported difficulty in identifying and assessing these impacts for any single study. This is understandable, as such impacts almost always result from a complex interplay of contributing factors; and there remains a need for alternative ways of conceptualizing and measuring longer term research ‘impacts’.

This study has a number of strengths and limitations. The strengths of this study were that impacts were assessed using multiple methods, including bibliometric analysis, interviews with researchers and end-users, and documentary checks. These data were triangulated and distilled into case summaries, which were used in a rigorous verification process involving independent assessments of impacts and a group panel assessment. The documentary checks lend confidence that the perspectives of the chief investigators and end-users were credible, while the verification panel process provides a well established and tested methodology for reaching expert consensus, and minimizing subjectivity of assessments. The end-users were purposefully sampled on the basis of having knowledge and/or experience of how the project findings had been used, and while this ensured they contributed relevant information, there was potential for some degree of social response bias, as some end-users may have been inclined to report positive impacts or over-inflate those impacts. We attempted to reduce social response bias by having researchers not previously involved in funded HPDRGS projects conducting the interviews and undertaking the analysis. The recall of impacts was somewhat uncertain for some projects from the early funding rounds, as these were conducted between 10 to 12 years ago. For one of the projects the end-users could not be identified. It is possible therefore that the impacts may have been underestimated for some of these older projects.

## Conclusions

This HPDRGS case series analysis provides new methods and insights into how intervention research projects influence policy and practice. Funded projects had variable impacts on policy and practice. Where impacts occurred they ranged from raising awareness of health interventions, identifying priority issues and target groups for interventions, underpinning new policies, and supporting and justifying existing policy and/or programs. The success of high impact projects was perceived in large part to be due to the nature and quality of the intervention itself (simple to understand, built in mechanisms for training and delivery), high quality research, champions who advocated for adoption, and active dissemination strategies. Our findings also highlight the need for strong partnerships between researchers and policy makers/practitioners to increase ownership over the findings and commitment to action.

## Abbreviations

CI: Chief investigators; HPDRGS: Health Promotion Demonstration Research Grants Scheme

## Competing interests

In order to minimize potential conflict of interest and response bias in the interviews, an independent research officer (RN) conducted the interviews with all of the chief investigators and end-users. This was particularly important as one of the authors (AJM) had previously administered the HPDRGS, and two others had been past recipients of its grants (AB, CR). Those involved in funded projects (AB, CR) declared their potential conflicts of interest at the verification panel meeting prior to any group assessment of impacts, they did not assess the impacts of their own projects, and did not contribute to final group assessments of their work.

## Authors’ contributions

AJM conceived the study and AJM, RL, LK, CR designed the methods. RN was responsible for conducting the interviews and collecting documentary sources. AJM and JB undertook analysis of the case study data, produced the case summaries. AJM drafted the manuscript. All authors contributed to data interpretation and have read and approved the final manuscript.
